# Editorial: Legal and ethical reforms for neurotechnological advancements in the global south: perspective and challenges

**DOI:** 10.3389/fnhum.2026.1985765

**Published:** 2026-09-29

**Authors:** Marietjie Botes, Andrea Lavazza, Kimberly Ray

**Affiliations:** 1 Pestilli Neuroscience Lab, Department of Psychology, University of Texas at Austin, Austin, TX, United States; 2 Department of Literary, Linguistic, and Philosophical Studies, Pegaso University, Naples, Italy

**Keywords:** cultural differences, Global South and globalization, neuroethics, neuroscience, neurotechnology

The global rules governing neurotechnology are being written now. The central question is not only how the mind should be protected, but who will have the power to define that protection, and whose histories, knowledge, and priorities will shape it.

Neurotechnologies promise new ways to restore communication, treat neurological and psychiatric conditions, and expand scientific knowledge of the brain. Their use also appears likely, perhaps inevitable, to extend to civilian domains, including the workplace, education, and recreation, as well as to security and military contexts. Across these domains, neurotechnologies may create unprecedented opportunities to infer, influence, or intervene in mental life. Questions of mental privacy, cognitive liberty, identity, autonomy, and equitable access therefore cannot be postponed until these technologies are mature and widely adopted, they must also be addressed in anticipation of likely future developments (Ligthart et al., 2023). Also, neither can governance be built on speculative harms, imprecise concepts, or assumptions presented as universal Lavazza and Giorgi, 2023. Our Research Topic aimed to bring these imperatives into conversation from the standpoint of the Global South, where the Global South broadly refers to countries and communities in Africa, Latin America, Asia, and Oceania that have historically occupied less powerful positions within the global economic and political order.

Although the Global South is not a single legal, cultural, or political space, the contributions to this Research Topic nevertheless converge on a shared warning, namely, that neurogovernance cannot be responsibly transplanted from one jurisdiction to another, or separated from colonial histories, material inequality, scientific power, and technological dependency (Botes et al., 2026). Their collective contribution is to move the debate beyond the simple question of whether new technologies require new individual rights. They ask a larger and more consequential set of questions: Who defines the harms that law should address? Whose conceptions of the mind and personhood become authoritative? Who participates in innovation? Who receives its benefits? And who controls the infrastructures through which neural data and cognitive life are increasingly mediated?

Two articles expose a productive tension at the center of current neurorights reform. In “What a NeuroRights legislation should not look like,” Borbón critiques the Latin American Parliament's Model Law for definitions and ethical concepts that are overly broad, scientifically uncertain, and liable to produce symbolic or misdirected regulation. Borbón favors targeted, evidence-based measures, supported where appropriate by existing rights, data-protection, and medical-device law, soft law, and international standards. By contrast, Moreira and Vilaça's comparative analysis of the Brazilian case concludes that Brazil's current framework, although relevant, remains insufficient for risks such as unauthorized neural monitoring, mental-state inference, cognitive manipulation, and misuse of neural evidence. Their proposal is not a wholesale imitation of Chile, but a multilayered approach combining reinterpretation of constitutional guarantees with carefully targeted statutory innovation. Read together, these articles reject both regulatory complacency and uncritical legal novelty. Their disagreement is not over whether protection is needed, but over how law can respond without outrunning scientific evidence or leaving genuine vulnerabilities unaddressed.

Zhukoff further complicates assessments of Chile's pioneering constitutional amendment. Her cognitive-sovereignty framework interprets neurorights not only as liberal protections of the individual, but also as a possible non-traditional security response by a technologically dependent state to disproportionate foreign influence over the mental and identity-related domains of its population. Chile's political history, bioethical architecture, and position as a consumer rather than producer of neurotechnology become essential to understanding what the amendment may do, irrespective of whether this function was explicit in its design.

The remaining contributions broaden the object of reform. Pyrrho's analysis of race, intelligence and genetics shows why equitable access, though vital, is insufficient. Concepts of intelligence, normality, ability, and enhancement have been shaped by colonialism, racism, eugenics, and biological determinism. Neurotechnology may reproduce these hierarchies when socially constructed categories as biological facts, Western ideals of optimization are universalized, or cognitive worth is reduced to economic productivity. Justice therefore requires scrutiny of the questions, classifications, datasets, and imagined futures that organize innovation.

Olalde-Mathieu and Licea-Haquet translate this critique into a positive epistemic program. Their account of cultural sensitivity, epistemic justice, and epistemic humility cautions that models derived largely from Western populations may not generalize across cultures, languages, and social contexts. Communities must participate in setting priorities, interpreting results, governing data, and designing technologies, rather than being treated merely as sources of participants or data. Inclusion is not only ethically required; it is a condition of scientific validity and clinical usefulness.

Saracini's account of cognitive sovereignty and neuro-justice connects these concerns to distributive justice and public health. Mental privacy offers little protection where people lack basic neurological care, infrastructures are externally controlled, or neural data and labor are extracted for benefits realized elsewhere. Her analysis also extends attention beyond devices that directly interface with the nervous system to algorithmic platforms and immersive systems capable of shaping attention, affect, and decision-making. This wider field demands accountability for both direct neural intervention and indirect cognitive modulation.

Across this discourse, these contributions identify four commitments for future governance: (1) conceptual precision and evidence of identifiable harm; (2) distributive access to beneficial technologies and basic care; (3) meaningful participation and epistemic authority for affected communities; and (4) protection against cognitive, technological, and data dependency. These commitments point toward governance that is layered, adaptive, and institutionally grounded. Existing constitutional and human-rights guarantees should be interpreted vigorously, sector specific regulatory regimes should be tested and strengthened, and new rights or statutes should be introduced where demonstrable gaps remain.

This Research Topic ultimately presents the Global South not as a passive recipient of universal neurorights, but as a generator of concepts and governance models capable of reshaping global neuroethics (Botes et al., 2025). Its central lesson is that protecting the mind requires more than drawing a legal boundary around neural data (Ray et al., 2026). It requires asking who defines the mind, whose knowledge counts, who benefits from innovation, and who controls the infrastructures through which cognitive life is increasingly mediated. Legal and ethical reform will be credible only when it answers all four questions together.

We look forward to continuing this conversation in Volume II of this Research Topic, and invite scholars, communities, policymakers, and innovators to help shape a future in which neurotechnology is governed not only where it is developed or commercialized, but wherever its consequences are experienced.
